# The Host-Pathogen interaction of human cyclophilin A and HIV-1 Vpr requires specific N-terminal and novel C-terminal domains

**DOI:** 10.1186/1472-6807-11-49

**Published:** 2011-12-20

**Authors:** Sara MØ Solbak, Victor Wray, Ole Horvli, Arnt J Raae, Marte I Flydal, Petra Henklein, Peter Henklein, Manfred Nimtz, Ulrich Schubert, Torgils Fossen

**Affiliations:** 1 From the Centre of Pharmacy, University of Bergen, N-5007 Bergen Norway; 2 Department of Chemistry, University of Bergen, N-5007 Bergen Norway; 3 Department of Structural Biology, Helmholtz Centre for Infection Research, D-38124 Braunschweig Germany; 4 Department of Molecular Biology, University of Bergen, N-5020 Bergen Norway; 5 Department of Biomedicine, University of Bergen, Jonas Lies vei 91, 5009 Bergen, Norway; 6 Institute of Biochemistry, Charité Universitätsmedizin-Berlin, D-10117 Berlin Germany; 7 Institute of Virology, Friedrich-Alexander-University, D-91054 Erlangen Germany

## Abstract

**Background:**

Cyclophilin A (CypA) represents a potential key molecule in future antiretroviral therapy since inhibition of CypA suppresses human immunodeficiency virus type 1 (HIV-1) replication. CypA interacts with the virus proteins Capsid (CA) and Vpr, however, the mechanism through which CypA influences HIV-1 infectivity still remains unclear.

**Results:**

Here the interaction of full-length HIV-1 Vpr with the host cellular factor CypA has been characterized and quantified by surface plasmon resonance spectroscopy. A C-terminal region of Vpr, comprising the 16 residues ^75^GCRHSRIGVTRQRRAR^90^, with high binding affinity for CypA has been identified. This region of Vpr does not contain any proline residues but binds much more strongly to CypA than the previously characterized N-terminal binding domain of Vpr, and is thus the first protein binding domain to CypA described involving no proline residues. The fact that the mutant peptide Vpr^75-90 ^R80A binds more weakly to CypA than the wild-type peptide confirms that Arg-80 is a key residue in the C-terminal binding domain. The N- and C-terminal binding regions of full-length Vpr bind cooperatively to CypA and have allowed a model of the complex to be created. The dissociation constant of full-length Vpr to CypA was determined to be approximately 320 nM, indicating that the binding may be stronger than that of the well characterized interaction of HIV-1 CA with CypA.

**Conclusions:**

For the first time the interaction of full-length Vpr and CypA has been characterized and quantified. A non-proline-containing 16-residue region of C-terminal Vpr which binds specifically to CypA with similar high affinity as full-length Vpr has been identified. The fact that this is the first non-proline containing binding motif of any protein found to bind to CypA, changes the view on how CypA is able to interact with other proteins. It is interesting to note that several previously reported key functions of HIV-1 Vpr are associated with the identified N- and C-terminal binding domains of the protein to CypA.

## Background

The viral protein R (Vpr) is encoded by the human immunodeficiency viruses types 1 and 2 (HIV-1/HIV-2), the simian immunodeficiency viruses (SIV) and primate lentiviruses [1,2]. This accessory protein facilitates transport of the pre-integration complex into the nucleus of non-dividing cells [3] and fulfils multiple functions in the viral life cycle, including increase of viral replication in non-dividing host cells, induction of G2 cell-cycle arrest [4,5], apoptosis [6,7] and transduction through cell membranes [8] (The multiple functions of Vpr are reviewed in [9-11]). Vpr interacts with several cellular factors, including the human peptidyl prolyl isomerase cyclophilin A (CypA) [12-14]. One of the main problems with existing antiretroviral therapy is that the viruses can develop drug resistance, which necessitates identification of new potential drug targets that overcome this problem. One approach that recently has received increased attention, is targeting host factors essential for the pathogen life cycle, rather than pathogen components directly [15-17]. CypA could be such a target as it is dispensable for cell viability [18,19], and viral replication of HIV-1 is determined to be effectively inhibited by use of selective inhibitors of CypA [20-25].

Recently, we investigated the interactions of CypA with the N-terminus of Vpr at atomic resolution [14]. Prolyl *cis*/*trans *isomerization of the highly conserved proline residues Pro-5, -10, -14 and -35 of Vpr are catalyzed by human CypA and require only very low concentrations of the isomerase relative to that of the peptide substrates. However, of the N-terminal peptides of Vpr investigated, only those containing Pro-35, which appears to be vital for manifold functions of Vpr, bind to CypA in surface plasmon resonance (SPR) biosensor experiments. Extensive analysis revealed that the binding region of N-terminal Vpr to CypA consisted of the heptapeptide motif RHFPRIW centered at Pro-35 [14].

The biological significance of the interaction of Vpr with CypA, including the extensively studied interaction of CypA with HIV-1 capsid (CA), that is crucial for viral replication [26,27], is still not completely understood (Reviewed in [25,28]). However, specific inhibitors of the prolyl *cis/trans *isomerase activity of CypA, such as cyclosporine A and SDZ-NIM811, inhibit HIV-1 replication [20-25] and a possible role of CypA in both entry and postentry events of the viral life cycle of HIV-1 has been indicated [29]. The interaction of HIV-1 Vpr with CypA is known to occur *in vitro *and *in vivo *[12,13,25], although the biological consequences thereof are disputed. The original reports concluded that CypA had significance for the *de novo *synthesis of Vpr, as the Vpr-mediated cell cycle arrest in HIV-1 infected T cells appeared to be eliminated in the absence of CypA activity [13]. More recently it has been suggested that the interaction of Vpr with CypA is independent of the ability of Vpr to induce G2 cell cycle arrest [12].

Independent of these data, previous studies have unambiguously shown the N-terminal residues from Ala-30 to Phe-34, which are adjacent to or incorporated in the N-terminal binding region of Vpr to CypA, comprised of ^32^RHFPRIW^38 ^centered at Pro-35, are crucial for the ability of Vpr to induce G2 cell cycle arrest [30-32]. Similarly, the ability of Vpr to induce G2 cell cycle arrest is also influenced by residues in the C-terminal region 71-90 [30,31,33-37].

All studies are in agreement that mutation of Pro-35 with Ala disrupts the interaction of Vpr with CypA [12,13], which is consistent with a conformational change in the hydrophobic core [38]. On the other hand, intriguingly, Ardon et al. reported that mutation of the C-terminal residue Arg-80 with Ala also prevented coimmunoprecipitation of Vpr with CypA [12]. However, the molecular explanation for the latter C-terminal mutation of Vpr to interfere with the interaction of the protein with CypA has remained elusive [12]. Mutation of Arg-80 with Ala may cause a change in the folded structure of full-length Vpr or, could in theory, alter the structure of a specific novel C-terminal binding region of Vpr to CypA. The Pro residues of HIV-1_NL4-3 _Vpr are located in the N-terminal domain of the protein, namely at positions 5, 10, 14 and 35. All previously determined binding domains of various proteins interacting with CypA have hitherto required at least one Pro residue as part of the binding region.

However, a limited number of studies have shown that CypA is able to bind to peptides that do not contain Pro residues through an alternative non-Pro-dependent binding region [39]. According to Demange et al., CypA exhibits two binding sites, of which the S1' site requires a Pro-containing substrate whereas the S2'-S3' site facilitates binding to peptides that do not contain Pro [39]. Saphire et al. reported that a Vpr-CypA fusion protein which has no isomerase activity and no capacity to bind to CA also rescues HIV-1 replication, which may indicate an important role of CypA associated with Vpr [40].

These facts, together with the observation of considerable amounts of CypA in virions [41] prompted us to study the interaction of Vpr with CypA in order to identify potential novel binding sites. This paper presents SPR binding studies of the interactions of CypA with full-length synthetic Vpr (*s*Vpr) and C-terminal *s*Vpr peptides. A novel specific non-Pro-containing C-terminal binding region of Vpr facilitating strong binding to CypA has been identified. The contribution of this novel C-terminal binding region together with the previously characterized N-terminal binding region RHFPRIW centered at Pro-35 has been demonstrated through characterization and quantification of the interaction of full-length Vpr with CypA.

## Results

To investigate the interaction between full-length Vpr and CypA in more detail, and to find an explanation for the previously undefined role of the C-terminus of Vpr with respect to this interaction, we performed interactions studies by SPR spectroscopy between highly pure recombinant CypA and full-length Vpr and C-terminal Vpr peptides. Additionally, possibly structural changes in the C-terminal region caused by substitution of Arg-80 by Ala were investigated by nuclear magnetic resonance (NMR) spectroscopy.

### Characterization of the interaction of C-terminal Vpr^75-90 ^with CypA using surface plasmon resonance spectroscopy

**Figure 1:**
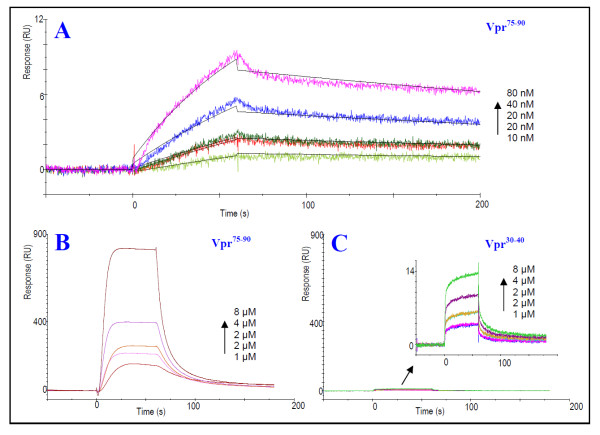
**Characterization of the specific interaction of C-terminal Vpr^75-90 ^with CypA and comparison with the interaction with N-terminal binding domain of Vpr**. (A) SPR sensorgrams for the specific interaction of synthetic Vpr^75-90 ^with CypA. The peptide was injected at concentrations 0-80 nM over CM5 chip immobilized with CypA to 918 RU. The SPR sensorgrams were fitted to a 1:1 (Langmuir) binding model (black line). (B-C) Comparison of interaction of Vpr^75-90 ^(B) and Vpr^30-40 ^(C) with CypA. The peptides were injected at concentrations ranging from 0-8 μM over CM5 chip immobilized to 180 RU with CypA. (B-C) illustrates the lower affinity of the previously characterized N-terminal binding domain of Vpr [14] to CypA compared with the novel detected C-terminal binding domain.

SPR data obtained for the interaction of C-terminal Vpr^75-90 ^with immobilized CypA revealed that the peptide binds strongly to CypA (Figure 1A, B). A comparison of sensorgrams obtained for the interactions of immobilized CypA with C-terminal Vpr^75-90 ^and the N-terminal peptide Vpr^30-40 ^containing the N-terminal binding region of Vpr to CypA [14], respectively, using the same analyte concentration (1-8 μM) and chip density of immobilized CypA (180 response units (RU)) (Figure 1B, C), showed that Vpr^75-90 ^has strongest binding to CypA. The magnitude of the SPR response is due to changes in the refractive index caused by mass changes, brought about by binding of analyte to ligand at the chip surface [42-44]. The SPR-angle response is converted to RU (1 RU = 0.0001°), which is equivalent to 1 pg/mm^2 ^of bound protein [44]. As Vpr^30-40 ^has a slightly lower molecular weight than Vpr^75-90^, a potential binding response would therefore be expected to be somewhat lower than for Vpr^75-90^, provided that the binding regions of the peptides have equal affinity for immobilized CypA. Nonetheless, the large difference in response observed for Vpr^75-90 ^compared with Vpr^30-40 ^(Figure 1B, C) can not be explained by the difference in molecular weight between the peptides.

Quantification of the kinetic data of the interaction of Vpr^75-90 ^with CypA was performed at low sample concentrations (10-80 nM) using a chip immobilized to 918 RU (Figure 1A and Table 1). The association and dissociation curves fitted well to a 1:1 binding model (Figure 1A), which was used to calculate the kinetic constants. The dissociation constant (K_D_) for the interaction was determined to be approximately 0.28 μM (Table 1).

**Table 1:** Estimated kinetic constants for binding of full-length HIV-1 Vpr and C-terminal Vpr peptides to CypA.

Peptide	Amino acid sequence	Model	Kinetic constants		
			K_D_(μM)	ka_1_(1/Ms)	kd(1/s)
Vpr^75-90^	GCRHSRIGVTRQRRAR	1:1	**0.28 ± **0.27	8.1 ± 6.1 × 10^4^	6.3 ± 4.6 × 10^-3^
Vpr^75-90 ^(R76Q, V83I, T84I)	GC**Q**HSRIG**II**RQRRAR	1:1	**4.7 **± 2.6	1.6 ± 0.1 × 10^4^	0.071 ± 0.037
Vpr^75-90 ^(R76Q, V83I, R80A, T84I)	GC**Q**HS**A**IG**II**RQRRAR	1:1	**2.7 **± 1.9	4.2 ± 0.8 × 10^4^	0.095 ± 0.061
Vpr^75-90 ^(R80A)	GCRHS**A**IGVTRQRRAR	1:1	**7.5 ± **2.6	3.8 ± 0.6 × 10^3^	0.027 ± 0.005
Vpr^69-78^	FIHFRIGCRH		*		
Vpr^75-84^	GCRHSRIGVT		*		
Vpr^81-90^	IGVTRQRRAR		*		
Vpr^87-96^	RRARNGASRS		*		
Vpr^1-96 ^(918 RU CypA)		BA		ka_1 _= 1.3 ± 1.0 × 10^4^ ka_2 _= 5.9 ± 1.7 × 10^-6^	kd_1 _= 1.8 ± 0.2 × 10^-3^ kd_2 _= 6.7 ± 3.8 × 10^-4^
Vpr^1-96 ^(918 RU CypA)		1:1	**0.31 **± 0.28	3.2 ± 1.2 × 10^4^	0.007 ± 0.005
Vpr^1-96 ^(180 RU CypA)		BA		ka_1 _= 5.8 ± 0.8 × 10^3^ ka_2 _= 1.6 ± 1.6 × 10^-4^	kd_1 _= 0.022 ± 0.025 kd_2 _= 7.3 ± 4.0 × 10^-4^
Vpr^1-96 ^(180 RU CypA)		1:1	**0.32 **± 0.14	1.6 ± 0.6 × 10^4^	4.22 ± 0.4 × 10^-3^

The data represent mean values ± S.E. of two individual experiments using four different concentrations containing duplicate of one concentration.

*K_D _not measurable by the Biacore instrument.

1:1, Langmuir binding

BA, Bivalent analyte

### Determination of the C-terminal binding region of Vpr to CypA

**Figure 2:**
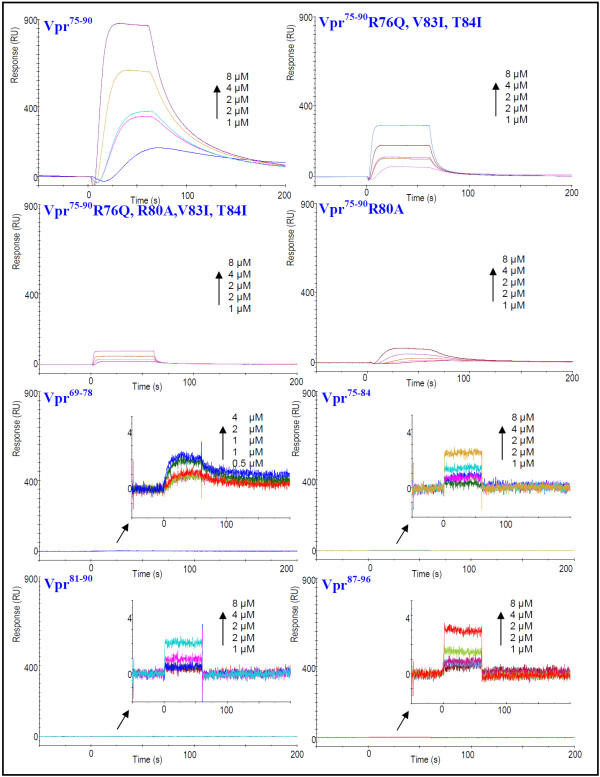
**Characterization of C-terminal Vpr peptides interacting with CypA**. SPR sensorgrams for synthetic Vpr^75-90^, the mutants Vpr^75-90 ^(R76Q, V83I, T84I), Vpr^75-90 ^(R76Q, V83I, R80A, T84I), Vpr^75-90 ^(R80A) and the shorter peptides Vpr^69-78^, Vpr^75-84^, Vpr^81-90 ^and Vpr^87-96 ^were analyzed for binding to immobilized recombinant CypA. The peptides were injected at concentrations ranging from 0-8 μM over CM5 chip immobilized to 150 RU with CypA.

To determine the exact C-terminal binding region of Vpr to CypA, the potential interaction of the partly overlapping C-terminal decapeptides Vpr^69-78^, Vpr^75-84^, Vpr^81-90 ^and Vpr^87-96 ^with CypA were analyzed by SPR spectroscopy. The sensorgrams revealed that these peptides interacted only weakly with CypA (Figure 2). Hence the complete C-terminal peptide Vpr^75-90^, comprising the 16 residues ^75^GCRHSRIGVTRQRRAR^90^, was required for maintaining the strong interaction with CypA.

### Mutation of Arg-80 with Ala significantly reduces the binding affinity

Previous studies show that the C-terminal region is important for most of the identified functions of Vpr, and attention has especially been directed to the arginine residues in the C-terminal region. As Arg-80 has previously been reported to be necessary for maintaining binding between Vpr and CypA [12], SPR experiments were performed on the mutant peptides Vpr^75-90 ^R80A, Vpr^75-90 ^(R76Q, V83I, T84I) and Vpr^75-90 ^(R76Q, V83I, R80A, T84I). A reduction in association was observed for all mutants compared with the *wild-type (wt) *peptide (Table 1). Interestingly, the peptides with mutation of Arg-80 (Vpr^75-90 ^R80A and Vpr^75-90 ^(R76Q, V83I, R80A, T84I)) showed the lowest binding response (Figure 2). The dissociation constant (K_D_) of the three mutants were of the same order of magnitude (10^-6^M) (Table 1). The highest dissociation constant was found for Vpr^75-90 ^R80A, indicating that this mutant exhibits the weakest binding to CypA. In summary, this implies an important function of Arg-80 in maintaining a strong interaction of the C-terminal binding region of Vpr to CypA, notwithstanding the fact that other residues appear to influence the interaction between Vpr and CypA to some extent. The fact that the decapeptide Vpr^75-84 ^fails to bind to CypA (Figure 2) clearly demonstrates that Arg-80 and its nearby surrounding residues are insufficient for the interaction to occur. Reduced binding affinity was also observed for the mutant peptide Vpr^75-90 ^(R76Q, V83I, T84I) where Arg-80 was conserved (Figure 2), demonstrating the importance of an intact 16 residue C-terminal binding domain of Vpr.

### Mutation of Arg-80 with Ala does not influence the weak secondary structure of the C-terminal binding domain of Vpr

**Figure 3:**
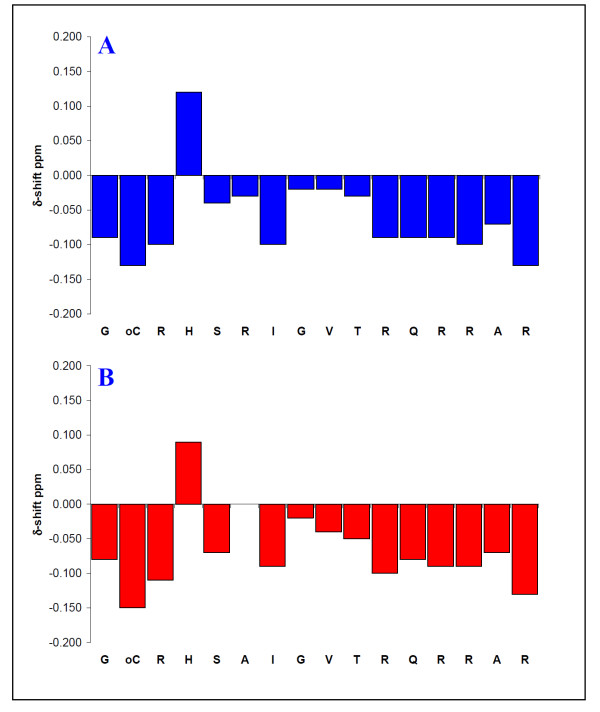
**Mutation of Arg-80 with Ala does not influence the secondary structure of Vpr^75-90^**. Chemical shift differences (ppm) of the α-protons between the experimental values and those for residues in a random coil for Vpr^75-90 ^(A) and *s*Vpr^75-90 ^R80A (B) in H_2_O-D_2_O 9:1 (v/v) containing 100 mM DPC-*d38 *at 300 K.

Previous NMR studies on Vpr [45] showed that the C-terminal binding domain of Vpr to CypA is a region that only exhibits weak secondary structure. To reveal whether or not the R80A mutation influences the secondary structure of the binding domain, NMR spectra of *wt *Vpr^75-90 ^and Vpr^75-90 ^R80A dissolved in aqueous solution in the presence of 100 mM dodecylphosphocholine (DPC) were recorded and chemical shift index (CSI) were performed. CSI are plots of the chemical shift differences of the α-protons relative to that of residues in a random coil and is commonly accepted to identify regions with secondary structure in peptides and proteins [46]. It has been shown experimentally that α-proton chemical shifts greater than 0.1 ppm relative to the random coil values are qualitative indicators of protein secondary structure [46]. A minimum of four adjacent residues with an upfield shift are indicative of an α-helix, whereas β-sheets require a minimum of three residues with downfield shifts [46]. CSI showed that the weak secondary structure of the binding region of Vpr is consistent with that previously reported by others in the literature [45] and is relatively uninfluenced by the R80A mutation (Figure 3).

### C-terminal Vpr binding to CypA confirmed by Isothermal titration calorimetry

**Figure 4:**
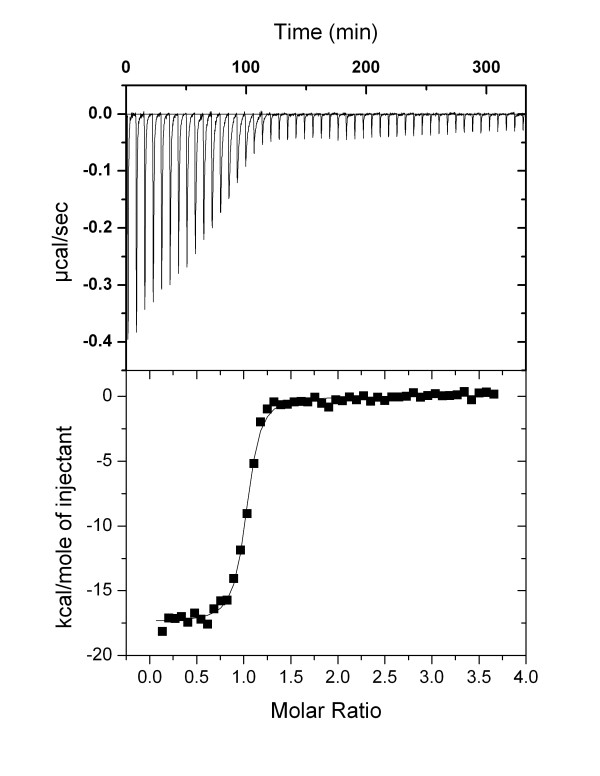
**ITC analysis of Vpr^75-90 ^binding to CypA**. Upper panel shows thermogram for the binding of Vpr^75-90 ^to CypA. Lower panel shows the corresponding binding isotherms where each point represents the integrated heat of the associated peak in the thermogram.

To independently confirm the binding of C-terminal Vpr^75-90 ^to CypA, and to verify the binding constant measured by SPR spectroscopy, isothermal titration calorimetry (ITC) titration was performed. The ITC analysis confirmed the high affinity interaction between Vpr^75-90 ^and CypA (Figure 4), and the mean dissociation constant from two independent titrations was found to be 0.09 μM (Table 2). The ITC experiment thereby verifies the approximate magnitude of the affinity of binding found by SPR spectroscopy (K_D _0.28 μM, Table 1).

**Table 2:** Estimated kinetic constants for binding of HIV-1 Vpr^75-90 ^to CypA by ITC

Titration	n	Ka(×10^7 ^M^-1^)	K_D_(μM)	ΔH(kcal/mol)	-TΔS(kcal/mol)
1	0.98	0.9	0.12	-17.4	7.0
2	1.00	1.7	0.06	-16.5	7.5
Mean values ± S.E		1.3 ± 0.4	0.09 ± 0.03	-16.9 ± 0.5	7.3 ± 0.3

### Full length Vpr binds strongly to CypA as a bivalent analyte

The N- and C-terminal binding regions of Vpr to CypA were accurately determined by SPR biosensor analysis of the interaction of N- and C-terminal peptides with a varying number of residues. These data encouraged characterization of the interaction of full-length Vpr with CypA using SPR spectroscopy.

**Figure 5:**
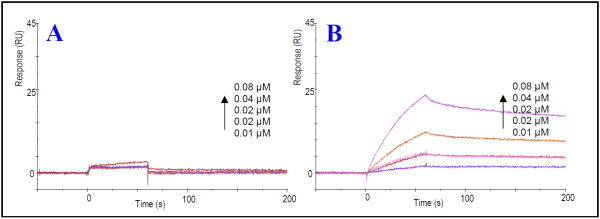
**Characterization of binding of full-length Vpr to CypA**. Characterization of CypA binding to full-lengthVpr at different chip concentrations of CypA. The interaction of Vpr^1-96 ^with immobilized recombinant CypA was analyzed using SPR biosensor system. Vpr^1-96 ^was injected over CM5 chips immobilized with different surface densities of CypA. Comparison of binding response curves detected for Vpr^1-96 ^injected at concentrations ranging from 0-80 nM over CM5 chip immobilized with 180 RU (A) and over chip immobilized with 918 RU CypA (B) shows an increase in response with increased CypA concentration immobilized.

**Figure 6:**
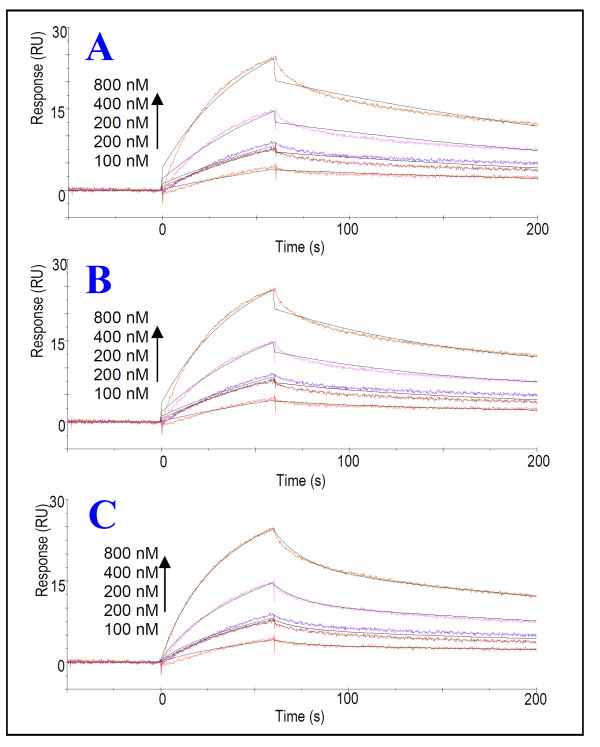
**Analysis of binding model for the full-length Vpr-CypA interaction**. SPR sensorgrams of the interaction of synthetic Vpr^1-96 ^with immobilized CypA. Vpr^1-96 ^was injected at concentrations 0-800 nM over CM5 chip immobilized with 180 RU CypA. The sensorgram was fitted to a 1:1 interaction model (A), heterogenous ligand kinetic model (B) and to a bivalent analyte kinetic model (C) using the biacore evolution program.

The SPR analysis of the interaction of full-length Vpr with CypA were performed with different analyte concentrations ranging from 10 nM to 8 μM and different ligand densities of CypA (150 RU, 180RU, 918 RU and 5003 RU). Comparison of the sensorgrams detected for Vpr^1-96 ^injected at concentrations ranging from 10-80 nM over a CM5 chip immobilized with 180 RU (Figure 5A), and over an analogous chip immobilized with 918 RU CypA (Figure 5B), shows a significant increase in response with increased CypA concentration immobilized on the chip. Thus, interaction analysis using lower concentrations of Vpr becomes accessible with application of higher chip densities of CypA. For full-length Vpr, concentrations ranging from 100 to 800 nM and a chip density of 180 RU CypA proved to be the optimum experimental condition for quantification of the interaction. Moreover, the sensorgrams obtained for the interaction of Vpr^1-96 ^with immobilized CypA revealed that the full-length protein binds strongly to CypA (Figure 6). As can be seen from the sensorgrams, the strength of the interaction seems primarily to be due to the slow dissociation phase (Figure 6). To quantify the interaction, the most appropriate binding model had to be determined. Two binding sites have been identified in Vpr, one N- and one C-terminal binding domain, which independently have been determined to bind to CypA by SPR spectroscopy. Consequently, the nature of the interaction between full-length Vpr and CypA is expected to be more complex than a 1:1 binding interaction. The presence of two binding sites of Vpr suggests that Vpr may act as a bivalent analyte for CypA. The question was whether these binding sites of Vpr bind at the same site at CypA or to two different sites, thus making CypA a heterogeneous ligand. Demange et al. suggested that there are two different and functionally independent subsites at CypA, namely a S1' proline substrate dependent subsite delineated by Met-61, Ala-101, Phe-113 and Leu-122 and a S2'-S3' Phe-pNA subsite surrounded by Ile-57, Phe-60, Trp-121 and Arg-148 that are able to bind to peptides that do not contain Pro residues [39].

As a consequence of our discovery that Vpr has one proline containing N-terminal CypA binding region and one non-proline containing C-terminal CypA binding region, these most likely bind to the S1' and S2'-S3' binding sites of CypA, respectively. Hence, CypA could be associated with both binding regions of Vpr simultaneously. Ardon et al. reported that both mutants Vpr P35N and Vpr R80A failed to coprecipitate with CypA [12], which implies that simultaneous binding of CypA to both binding sites of Vpr is required for CypA to bind to full length Vpr.

The SPR sensorgrams of the interaction of full-length Vpr with immobilized CypA gave an excellent fit to the bivalent analyte model. This strongly supports simultaneous binding of CypA with the N-terminal Pro-containing binding region and the C-terminal non-Pro binding region of Vpr, involving the S1' (Pro-dependent) and S2'-S3' (Pro-independent) binding sites of CypA, respectively (Figure 6). The stoichiometry of the interaction is 1:1 and as an estimate, the interaction was quantified from the simple 1:1 binding model. The sensorgrams of Vpr^1-96 ^were fitted to a 1:1 binding model by the Biacore evaluation software, and kinetic constants were determined (Figure 6, Table 1). The dissociation constant (K_D_) was determined to be approximately 320 nM.

## Discussion

Recently we determined that the N-terminal binding region of Vpr to CypA is comprised of the heptapeptide ^32^RHFPRIW^38 ^centered at Pro-35 [14]. Mutation of the central Pro residue (P35A) causes a loss of binding through disruption of the Pro-dependent N-terminal heptapeptide binding region. In the context of the full length molecule this mutation also results in merging of helix 1 and 2, which in turn causes a substantial change in the hydrophobic core of the protein as the anti-parallel folded structure of the N-terminal and central helices is no longer possible [38].

In this paper we have characterized a C-terminal region of Vpr, comprising the 16 residues ^75^GCRHSRIGVTRQRRAR^90^, with high binding affinity for CypA. This region of Vpr does not contain any Pro residues, but binds much more strongly to CypA than to the N-terminal binding region (Figure 1). The fact that the mutant peptide Vpr^75-90 ^R80A binds considerably weaker to CypA than the *wt *peptide (Figure 2) confirmed that Arg-80 is a key residue in the C-terminal binding region. Arg-80 is considered to be located in proximity to, but not included in, the well-defined helix 3 (residues 55-77) of the structured protein formed under membranous conditions at physiological pH [45]. The secondary structure NOEs of residues 78-90 of full-length Vpr found by Morellet et al. [45] are mainly restricted to those between NH-NH_(i, i+1)_, in addition to a few Hα-NH_(i, i+2) _and Hα-NH_(i, i+3)_, indicating that these residues comprise a region with a relatively weak α-helical structure. Indeed, our NMR data of *wt *Vpr^75-90 ^and Vpr^75-90 ^R80A confirmed this interpretation and revealed that the propensity for weak helical structure of the C-terminal binding region is essentially unaffected by the mutation of Arg-80 to Ala (Figure 3). Thus, the mutation prevents binding of Vpr to CypA through a local change in the C-terminal binding region rather than any change in the tertiary structure of Vpr.

In aqueous solution Vpr is present as high order aggregates (~decamers) with a lower percentage of higher multimers [8]. According to Fritz et al. oligomerization is mediated by the Vpr hydrophobic core but not by the flexible N- and C-terminal domains [47]. The C-terminal binding domain of Vpr to CypA is located beyond the residues that have been shown experimentally to be involved in oligomerization of Vpr. In agreement with this we used the statistical mechanics algorithm TANGO, which identifies aggregation-prone regions of peptides and denatured proteins using a set of balanced physico-chemical parameters [48,49]. According to the TANGO algorithm, a score of ≤ 0.02% indicates no aggregation, 0.02-5.0% indicates moderate aggregation, and ≥ 5.0% indicates high aggregation propensities. Application of this program predicted a region of 16 residues (Ala-55 to Ile-70) populating the oligomerization state to more than 5% per residue (5.09 - 35.56% per residue). Four further residues were predicted with much lower score, namely Thr-53 (1.30%), Trp-54 (3.50%), His-71 (0.84%) and Phe-72 (0.80%). The fact that the dissociation constant of full-length Vpr is in the same order of magnitude as the dissociation constant of the C-terminal binding domain Vpr^75-90 ^does not indicate that oligomerization of Vpr influences the interaction with CypA significantly.

Previous studies have shown that the mutants Vpr P35A and Vpr R80A were key residues for coimmunoprecipitation of Vpr and CypA, and when replaced abrogated the coimmunoprecipitation to CypA [12]. Although, these authors concluded that residues beyond Vpr^1-40 ^also are important for binding to CypA, no further explanation was provided. Our data independently confirm the importance of these residues for the interactions and indicate that the C-terminal binding is quantitatively stronger than the N-terminal binding to CypA (Figure 1). However, the fact that the decapeptide Vpr^75-84 ^fails to bind to CypA (Figure 2) clearly demonstrates that Arg-80 and its nearby surrounding residues are insufficient for the interaction to occur. Reduced binding affinity was also observed for a mutant Vpr^75-90 ^peptide where Arg-80 was conserved, demonstrating the importance of an intact 16 residue C-terminal binding domain of Vpr.

A simultaneous interaction of both Vpr sites with CypA was suggested as SPR sensorgrams of full-length Vpr interacting with immobilized CypA gave an optimal fit with a bivalent analyte model (Figure 5). Taken together, these data indicate that a simultaneous binding of the N- and C-terminal domains is required for full-length Vpr to interact with CypA. The dissociation constant (K_D_) of full-length Vpr was found to be approximately 0.32 μM (Table 1), which implies that the Vpr-CypA interaction may be stronger than the well characterized interaction of HIV-1 CA with CypA, which has been determined previously by SPR spectroscopy (K_D _16 ± 4 μM) [50]. This suggests that the CypA-Vpr interaction has a functional role in the relationship between the host and pathogen.

**Figure 7:**
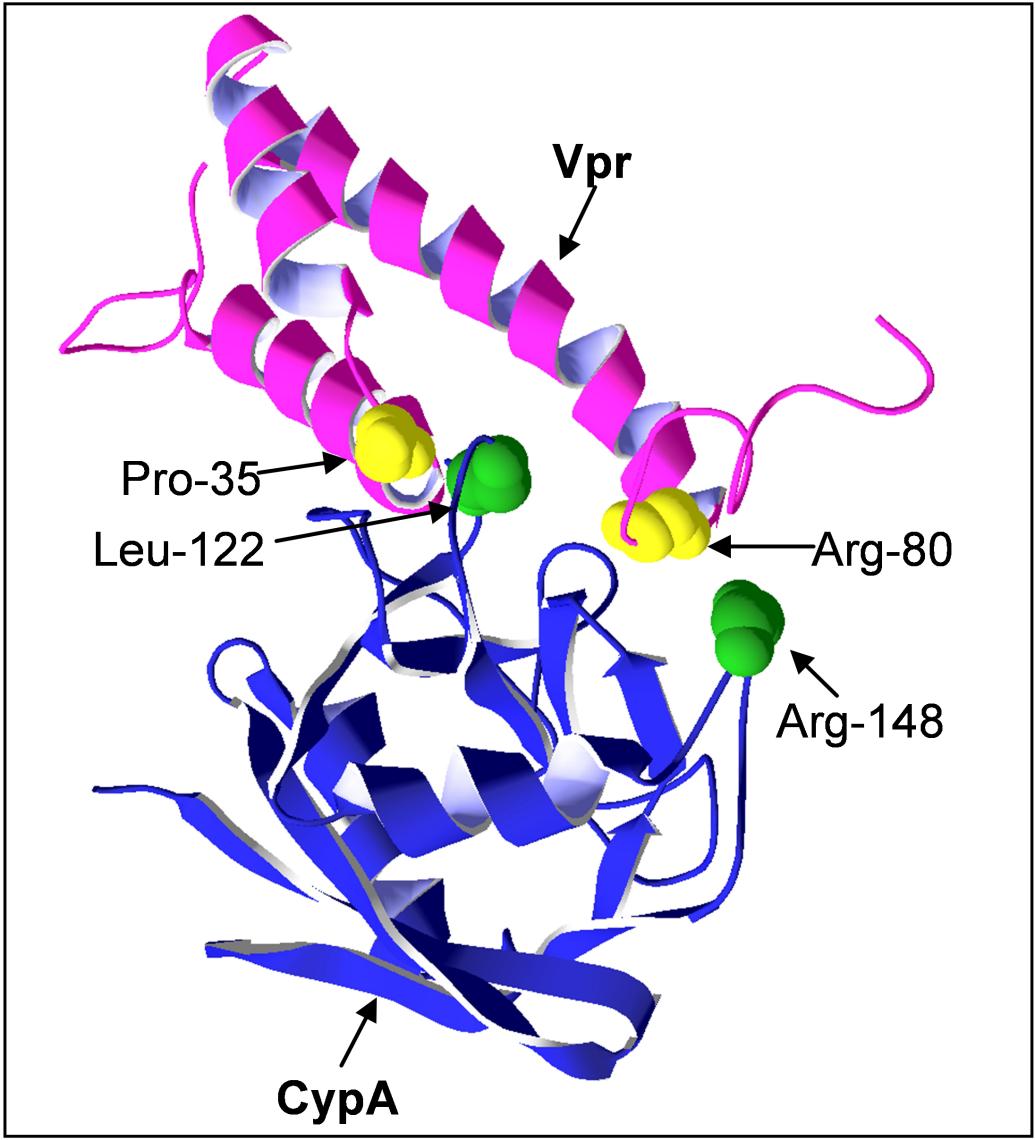
**Cartoon of the complex of Vpr and CypA**. The cartoon shows the 30 KDa complex of CypA with full-length Vpr where selected residues of the cooperative N- and C-terminal binding domains of Vpr and the two binding sites of CypA suggested by Demange et al. are high-lighted [30]. Residues Pro-35 and Arg-80 of Vpr are highlighted in yellow. Leu-122 of the Pro-dependent S1' subsite and Arg-148 of the Pro-independent S2'-S3' subsite of CypA are highlighted in green [30]. The structures of CypA and Vpr used in the model of the complex are derived from the pdb files of the X-ray structure of CypA at 1.63 Å resolution [51] and the NMR structure of Vpr obtained in aqueous 30% acetonitrile solution at low pH [45]. At physiological pH Vpr is mainly structured in a membranous environment and exhibits a more flexible structure under hydrophilic solution which is similar to the experimental conditions used in the SPR experiments (aqueous buffer pH 7.4) [8,53].

The access to detailed information of the interaction of full-length Vpr with CypA, based on the experimental characterization of the N- and C-terminal binding domains of Vpr, now allows us to visualize the interaction of full-length Vpr with CypA. As prerequisites, we have used the NMR structure of Vpr [45] and X-ray structure of CypA [51] as rigid units, together with knowledge of the key residues of the Pro-dependent and Pro-independent binding domains of CypA [39]. A cartoon illustrating the Vpr-CypA complex based on these parameters was generated with the ZDOCK algorithm [52] and is shown in Figure 7. This crude model suggests that a folded Vpr structure is required to provide N- and C-terminal binding regions sufficiently close in space in the Vpr-CypA complex (Figure 7) for the cooperative interaction with the two binding sites of CypA. Currently one should keep in mind that the model has its limitations but clearly rationalizes the findings presented here. The structure of Vpr used, which is the potentially variable/flexible component of the model, is the limiting structure at low pH in 30% aqueous acetonitrile [45]. However, a more flexible structure of Vpr, which would be expected under the hydrophilic conditions at physiological pH [8,53] used in the Biacore experiments performed to characterize the interaction, would be beneficial for improving the model and, thus, provide more accurate details of the interactions of the Vpr-CypA complex at the atomic level.

Most residues of the N- and C-terminal binding domains of Vpr are highly conserved among HIV-1 strains (with the exception of Ile-37 of the N-terminal binding domain and Arg-77, Val-83, Thr-84, Arg-85, Gln-86 and Ala-89 of the C-terminal binding [54]), indicating that maintenance of these structural regions of the protein is important for the viral life cycle.

**Figure 8:**
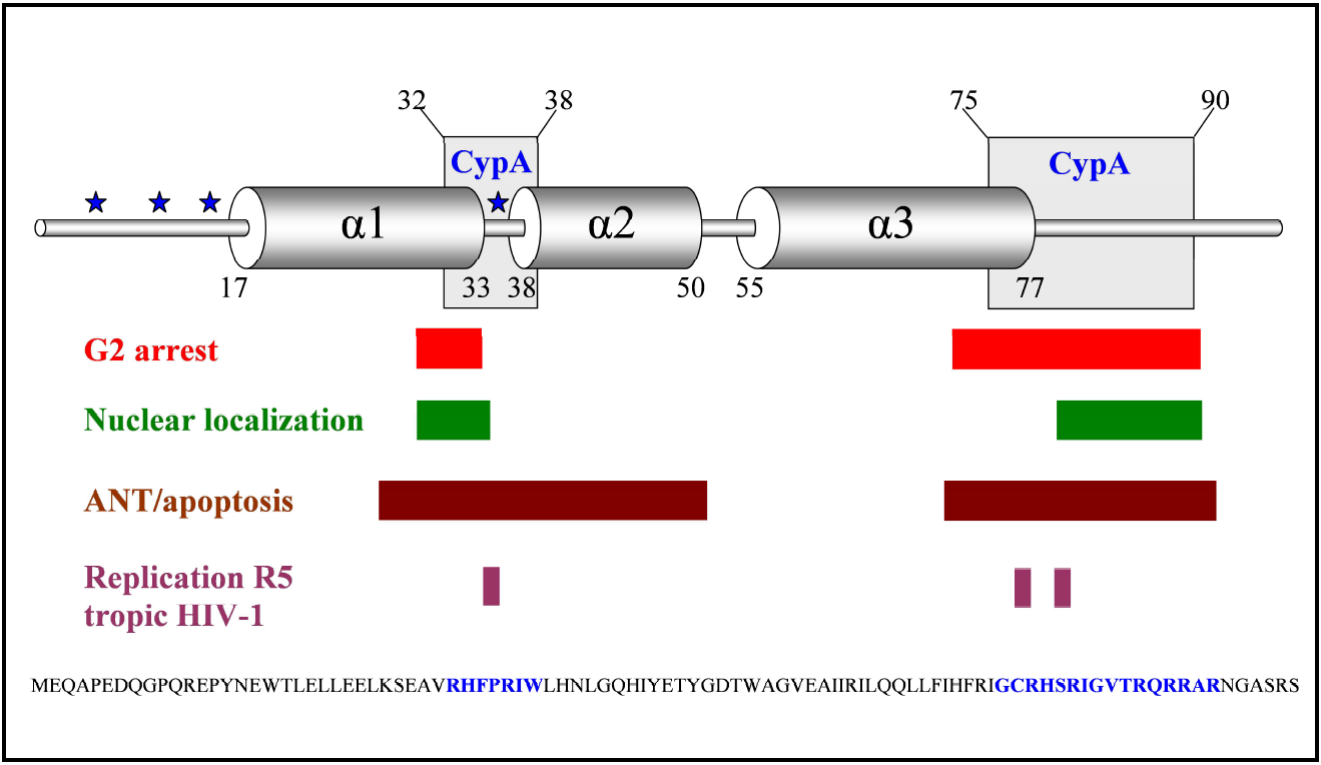
**Previously determined biological functions associated with the N- and C-terminal binding domains of Vpr to CypA**. Linear structure of full length Vpr with incorporated biological functions connected to the determined N- and C-terminal binding regions of Vpr to CypA including G2 cell cycle arrest [30-34,36,71], nuclear localization of the protein [30,31,68-70], ANT/apoptosis [55-63], and replication of R5 tropic HIV-1 [38,67]. Residues belonging to the N- and C-terminal binding regions of Vpr to CypA are labeled in blue.

The N-terminal Vpr binding domain is located between helix 1 and 2, and the C-terminal Vpr binding domain is located in an arginine-rich region in proximity to the third α-helical domain of Vpr (Figure 7). Although the biological importance of the binding of Vpr to CypA remains elusive, multiple functions of Vpr are connected to the two regions of the protein which bind cooperatively to CypA, including apoptosis [55-63], reverse transcriptase (RT) activity [36,64-66], replication of R5 tropic HIV-1 [38,67], nuclear localization of the protein [30,31,68-70], G2 cell cycle arrest [30-36,71] and binding of Vpr to DNA and RNA, which is linked to the ability of activating the ATR (ataxia-telangiectasia and Rad3-related) pathway leading to G2 arrest [72-75] (Figure 8). The importance of both domains to achieve binding of full-length Vpr to CypA suggests that the biological relevance of the interaction may be associated with other functional interactions of Vpr involving both the N- and C-terminal regions (Figure 8). Previous studies have presented contradictory results regarding the possibility that the interaction of Vpr with CypA is involved in induction of G2 arrest or that it has any effect on Vpr expression [12,13]. However, other studies have independently shown that Vpr induced G2 arrest is dependent on residues belonging to both the N- and C-terminal binding regions of Vpr to CypA [30-36,71]. Residues within the C-terminal domain of Vpr including Arg-80, which was found essential for retaining a strong binding to CypA in this study, are also considered to be important for increasing RT activity [36] and Vpr induced apoptosis [56,60,61]. Furthermore, residues in the N-terminal binding region of Vpr are important for Vpr induced apoptosis (Figure 8) [57,58,62], indicating that, both the N- and C-terminal CypA binding domains are involved in this function of Vpr. Moreover, the nuclear localization of Vpr and replication of R5 tropic HIV-1 are related to residues included in both N- and C-terminal CypA binding domains of Vpr (Figure 8). The identification of N- and C-terminal binding domains of Vpr, which cooperatively bind to CypA (Figure 7), should encourage further investigations into the biological relevance of the interaction of Vpr with the host cellular factor CypA, as it is likely that this strong interaction is of importance in the viral life cycle of HIV-1.

## Conclusions

CypA may represent a potential key molecule in future antiretroviral therapy since inhibition of CypA suppresses HIV-1 replication. All previous studies of protein-protein interactions involving human CypA have hitherto been limited to Pro-containing substrates. The fact that a non-proline-containing 16-residue region of a protein is able to bind with high affinity to CypA, presented for the first time in this manuscript, changes the view on how CypA is able to interact with other proteins. It is interesting to note that several previously reported key functions of HIV-1 are associated with the identified N- and C-terminal binding domains of the protein to CypA.

## Methods

### Peptide synthesis

The synthesis, purification and molecular characterization of synthetic Vpr^1-96 ^(*s*Vpr), the C-terminal peptides Vpr^75-90 ^, Vpr^69-78^, Vpr^75-84^, Vpr^81-90 ^and Vpr^87-96 ^and the mutants Vpr^75-90 ^(R76Q, V83I, T84I), Vpr^75-90 ^(R76Q, V83I, R80A, T84I), Vpr^75-90 ^(R80A) were performed as described in detail elsewhere [8,53] and the purities were checked by HPLC, MALDI-MS and positive ion ESI-MS [14]. The mass spectrum of *s*Vpr exhibited a significant M-18 peak corresponding to loss of a water molecule from the intact protein during synthesis. As tandem mass spectrometry **(**MS/MS) analysis revealed that the water molecule was eliminated from residues 6 or 7, belonging to a region that has been experimentally shown not to bind to CypA [14], the protein was considered suitable for our studies.

### Cyclophilin A (CypA)

The production and purification of recombinant human CypA has been described previously [14].

### Nuclear magnetic resonance spectroscopy

2D ^1^H Total correlation spectroscopy (TOCSY) and nuclear Overhauser enhancement spectroscopy (NOESY) NMR experiments were performed at 600.13 MHz on a Bruker Avance 600 MHz instrument equipped with an UltraShield Plus magnet and a triple resonance cryoprobe with gradient unit. Individual samples were dissolved in 600 μl 100 mM aqueous DPC-*d38 *micelles 10% D_2_O (v/v) at concentrations between 1-2 mM. The 2D NMR experiments were performed at 300 K without spinning with mixing times of 110 ms for the TOCSY experiments and 250 ms for the NOESY experiments. Efficient suppression of the water signal was achieved by application of excitation sculpting in the 1D ^1^H and the 2D ^1^H TOCSY and NOESY NMR experiments [76]. ^1^H signal assignments of the NMR spectra were achieved by identification of the individual spin systems in the 2D ^1^H TOCSY spectra, combined with observations of sequence-specific short-distance crosspeaks (Hα-HN i, i+1) in the 2D ^1^H-^1^H NOESY spectra [53,77]. Readily recognizable spin systems were used as starting points for correlation of the individual spin systems observed in the TOCSY and NOESY spectra with individual residues in the peptide sequences. Acquisition of data, processing and spectral analysis were performed with Bruker Topspin 1.3 software.

### Surface plasmon resonance spectroscopy

SPR [42-44] measurements were performed at 25°C on a Biacore T100 instrument (Biacore AB, Uppsala, Sweden) equipped with CM5 research-grade sensor chips. CypA was immobilized to 150, 180, 918 and 5003 RU, using standard amine-coupling chemistry. The reference flow cells were treated correspondingly except for CypA immobilization. The synthetic full length Vpr, as well as fragments and mutants thereof, were dissolved at four different concentrations in the running buffer (HBS-EP buffer pH 7.4; 10 mM HEPES, 150 mM NaCl, 3.4 mM EDTA and 0.005% surfacant). The samples were injected over the flow cells at a flow rate of 30 μl/min. Data were collected at 2.5 Hz during 60 s association and 180 s or 240 s dissociation phases, and were automatically corrected for bulk buffer effects and non-specific binding of Vpr peptides to the chip matrix. All SPR data was acquired from two individual series of experiments using four different analyte concentrations with a duplicate injection of one of the individual concentrations.

### Analysis of biosensor data

Affinity, association and dissociation rate constants were obtained from sensorgrams by the Biacore T100 evaluation software version 2.0.1 in accordance with the global curve fit model. Sensorgram data for the four different concentrations were fitted to several binding models including 1:1 (Langmuir) binding model (A+B ↔ AB), two-state reaction (conformational change) model (A+B ↔ AB ↔ AB*), heterogeneous ligand (HL) model (interaction one: A+B1 ↔ AB1; interaction two: A+B2 ↔ AB2) and bivalent analyte (BA) model (A+B ↔ AB; AB+B↔ABB). Kinetic constants were calculated for the best fitted model.

### Isothermal titration calorimetry

ITC was performed in a VP-ITC titration calorimeter (MicroCal Inc.). CypA and Vpr^75-90 ^were prepared in filtered HBS-EP buffer pH 7.4 and the samples were degassed prior to titration. Two individual titrations were performed by adding 14.8 μM and 11 μM CypA, respectively to the sample cell and titrated with a 210 μM stock solution of Vpr^75-90^. CypA samples were subjected to 30-50 injections (5-10 μL) of the Vpr^75-90 ^peptide with a 360 or 420 s interval between each injection at 25°C. The mean of the heat from the last 5 injections was subtracted from the raw data to correct for experimental heat dilution. The binding isotherms were fitted to a one site binding model using the Origin v7 software (OriginLab). Chi-square minimization was performed iteratively to obtain the best-fit parameters.

### Docking analysis of the Vpr-CypA complex

A cartoon of the Vpr-CypA complex was generated based on the detailed experimental characterization of the N- and C-terminal binding domains of Vpr using the ZDOCK algorithm [52]. The X-ray structure of CypA (pdb entry 3K0N) [51] and the NMR structure of Vpr (pdb entry 1M8L) [45] were used as rigid units, together with knowledge of the key residues of the Pro-dependent and Pro-independent binding domains of CypA.

## Authors' contributions

SMØS, VW and TF participated in planning the experimental work. SPR measurements were performed by SMØS, OH and analyzed by SMØS, TF and AJR. PH and PH synthesized the peptides. ITC measurement and analysis were performed by MIF and SMØS. US provided the highly pure recombinant CypA that was used in the experiments. MN performed and analyzed MS data. Docking analysis was performed by AJR. SMØS and TF planned and performed the NMR studies and SMØS, VW and TF wrote the manuscript. All authors read and approved the final manuscript.
